# El *continuum* de violencia en Centroamérica, México y EEUU y la salud mental de adolescentes migrantes no acompañados: estudio exploratorio en las ciudades de México y San Francisco

**DOI:** 10.1590/0102-311XES118525

**Published:** 2026-03-16

**Authors:** Tonatiuh Tomás González-Vázquez, Martha Decker, Silvana Larrea-Schiavon, Maria del Pilar Torres-Pereda

**Affiliations:** 1 Centro de Investigación en Sistemas de Salud, Instituto Nacional de Salud Pública, Cuernavaca, México.; 2 Department of Epidemiology and Biostatistics, University of California, San Francisco, United States.; 3 School of Public Health, University of California, Berkeley, United States.

**Keywords:** Menores No Acompañados, Migración Humana, Violencia, Salud Mental, Unaccompanied Minors, Human Migration, Violence, Mental Health, Menores Desacompanhados, Migração Humana, Violência, Saúde Mental

## Abstract

La migración irregular de niñas, niños y adolescentes no acompañados de Centroamérica hacia México y Estados Unidos ha aumentado en la última década. Estos adolescentes enfrentan un *continuum* de violencia en el origen, tránsito y destino, que impacta su salud mental y bienestar. Este estudio analiza las violencias experimentadas por niñas, niños y adolescentes no acompañados en sus comunidades de origen, durante el tránsito migratorio y en el destino, explorando los problemas de salud mental asociados y las estrategias de afrontamiento implementadas. Se realizó un estudio cualitativo binacional (junio 2022-junio 2023) con entrevistas semiestructuradas a 20 niñas, niños y adolescentes no acompañados de origen centroamericano y 14 informantes clave en México y Estados Unidos. Niñas, niños y adolescentes no acompañados reportaron múltiples violencias en el origen, durante el tránsito y en el destino. Estas experiencias generaron consecuencias emocionales, cognitivas y conductuales consistentes con estrés migratorio, incluyendo ansiedad, síntomas depresivos, ideación suicida y dificultades de adaptación. A pesar de ello, los adolescentes desplegaron estrategias de afrontamiento como migrar para escapar de la violencia, denunciar a perpetradores, conformar redes de apoyo con pares y organizaciones de la sociedad civil, continuar estudios y reiniciar la vida familiar en destino. Los hallazgos evidencian la exposición de niñas, niños y adolescentes no acompañados a un *continuum* de violencias estructurales y cotidianas que afectan su salud mental y desafían la protección estatal. Sin embargo, también muestran su capacidad de resiliencia y agencia. Se requiere un abordaje integral en políticas públicas que reconozca estas experiencias, garantice atención en salud mental y asegure el interés superior de la niñez migrante.

## Abstract (en)

The irregular migration of unaccompanied girls, boys, and adolescents from Central America, Mexico, and the United States has increased over the past decade. These individuals face a continuum of violence at the origin, path, and destination of their trajectories that affects their mental health and well-being. This study investigates the violence suffered by these unaccompanied girls, boys, and adolescents in their communities of origin, migratory journey, and final destination, exploring the associated mental health problems and implemented coping strategies. This binational qualitative study was carried out (June 2022-June 2023) with semi-structured interviews with 20 unaccompanied girls, boys, and adolescents from Central America and 14 key informants in Mexico and the United States of America. Unaccompanied girls, boys, and adolescents described multiple forms of violence in their place of origin, during the path, and final destination. These experiences generated emotional, cognitive, and behavioral consequences consistent with migratory stress, including anxiety, depressive symptoms, suicidal ideation, and difficulties in adapting. However, adolescents used coping strategies such as migrating to escape violence, denouncing perpetrators, consolidating support networks with peers and civil society organizations, continuing their studies, and starting family life again at their destination. The findings show the exposure of unaccompanied girls, boys, and adolescents to a continuum of structural and daily violence that affects their mental health and challenges government support. However, they also show their capacity for resilience and action. A comprehensive public policy approach is needed that acknowledges these experiences, guarantees mental health care, and ensures the best interest of these young migrants.

## Abstract (pt)

A migração irregular de garotas, garotos e adolescentes desacompanhados da América Central, México e Estados Unidos tem aumentado na última década. Estes indivíduos enfrentam um *continuum* de violência na origem, caminho e destino de suas trajetórias, afetando a saúde mental e bem-estar. Este estudo investiga a violência sofrida por estes garotos, garotas e adolescentes desacompanhados em suas comunidades de origem, durante o percurso migratório e o destino final, explorando os problemas de saúde mental associados e as estratégias de enfrentamento implementadas. Foi realizado um estudo qualitativo binacional (junho de 2022-junho de 2023) com entrevistas semiestruturadas com 20 garotas, garotos e adolescentes desacompanhados oriundos da América Central e 14 informantes-chave no México e Estados Unidos. Garotas, garotos e adolescentes desacompanhados descreveram múltiplas formas de violência no seu local de origem, durante o caminho e no destino final. Estas vivencias geraram consequências emocionais, cognitivas e comportamentais consistentes com estresse migratório, incluindo ansiedade, sintomas depressivos, ideação suicida e dificuldades em se adaptar. Contudo, os adolescentes utilizaram estratégias de enfrentamento como migração para escapar da violência, denuncia de perpetradores, consolidar redes de apoio com pares e organizações da sociedade civil, continuar os estudos e recomeçar a vida em família no destino. Os achados evidenciam a exposição dos garotas, garotos e adolescentes desacompanhados a um *continuum* de violências estruturais e cotidianas que afetam sua saúde mental e desafiam o suporte governamental. Contudo, também mostram sua capacidade de resiliência e ação. É necessário uma abordagem integral de políticas públicas que reconheça estas vivências, garanta atenção em saúde mental e assegure o interesse destes jovens migrantes.

## Introducción

Las migraciones irregulares de niñas, niños y adolescentes por México han tenido un ascenso en los últimos años con: 11.262 eventos en el 2020, 75.592 en el 2021, 71.207 en el 2022 y 113.542 en el 2023. La proporción de niñas, niños y adolescentes no acompañados respecto al total de niñas, niños y adolescentes presentados a las autoridades migratorias fue de 42% en el 2020, 19% en el 2021 y 2022 y 6% en el 2023 ^1^.

De los años 2020 al 2023, el 84% o más de las niñas, niños y adolescentes no acompañados eran originarios de Guatemala, Honduras y El Salvador. En el 2022, entre los que provenían de estos países (11.746), el 71% eran hombres y el 29% mujeres ^2^ ^,^ ^3^ ^,^ ^4^ ^,^ ^5^. Respecto a Estados Unidos (EEUU) las niñas, niños y adolescentes no acompañados aumentaron de 13.625 en el año 2012, a 118.938 en el 2023, para este año, la mayoría provenían de Guatemala (42%), Honduras (28%) y El Salvador (9%), el 61% eran hombres y el 38% mujeres ^6^.

Uno de los principios fundamentales de la Convención de las Naciones Unidas sobre los Derechos del Niño es el interés superior de las niñas, niños y adolescentes. En su Artículo 3, establece que en todas las medidas concernientes a ellos que adopten las instituciones públicas o privadas de bienestar social, los tribunales, las autoridades administrativas o los órganos legislativos, deberá considerarse como una consideración primordial su interés superior. Debido a la ambigüedad del concepto, se han dado diversas interpretaciones, siendo criticado de paternalista, al entenderlo como obligaciones dirigidas a las instituciones estatales, a los padres, madres y personas adultas. En el 2013, el Comité de los Derechos del Niño efectuó una interpretación jurídica en donde se ve a las niñas, niños y adolescentes no solamente como sujetos de derecho, sino que también son sujetos sociales, para quienes los derechos tienen que tener sentido y ser relevantes en su vida cotidiana. Se define el término de interés superior: (a) como derecho sustantivo (con aplicabilidad inmediata y reclamable judicialmente); (b) como principio jurídico interpretativo fundamental, y (c) como norma de procedimiento. Señalando que este artículo ha de aplicarse de tal forma que todos los derechos establecidos en la Convención se observen de la misma manera, cumpliéndose sus derechos de participación, donde las niñas, niños y adolescentes tengan posibilidad de participar en la determinación de lo que es su interés superior y en su implementación ^7^.

Si bien Guatemala, Honduras, El Salvador, Nicaragua y México han ratificado esta Convención y reconocido en sus legislaciones nacionales el interés superior de la niñez, se conoce poco sobre las implicaciones prácticas que esto tiene para las niñas, niños y adolescentes no acompañados antes de migrar, durante su tránsito por Centroamérica y México y/o en su lugar de destino (México o EEUU). Por su parte, EEUU se retiró del Pacto Mundial sobre Migración en 2018 y no ratificó la Convención ^8^ ^,^ ^9^.

Múltiples causas llevan a que las niñas, niños y adolescentes migren de Centroamérica, entre otras están la violencia, el abuso, la trata de personas, la reunificación familiar y la extrema pobreza. Las violencias pueden ser ejercidas por el crimen organizado, los funcionarios públicos (fuerzas armadas, autoridades migratorias), y la familia, tanto en el origen, como en los países de tránsito y destino, generando un *continuum* de violencia. Este concepto se ha utilizado con poblaciones migrantes latinoamericanas en tránsito. En el caso de las mujeres, considera que la violencia de género se basa en condiciones de desigualdad que las posiciona en desventaja respecto a los hombres, bajo un esquema patriarcal. Se explica cómo dinámicas entre desiguales, que producen violencias sistemáticas a lo largo del ciclo de vida y no como acciones individuales y aisladas. Por su parte, en el caso de las violencias contra los jóvenes centroamericanos que transitan o radican en México se identifica un *continuum* de violencias legales, paralegales, estatales, de mercado, pero también familiares y de pares, que vulnerabilizan la existencia de los jóvenes y el ejercicio de sus derechos ^10^ ^,^ ^11^.

Los eventos traumáticos experimentados por las poblaciones migrantes en el trayecto y destino, provocan afectaciones emocionales, cognitivas y conductuales, denominadas como estrés migratorio ^12^. Distintas revisiones sistemáticas de literatura han documentado sus efectos negativos en la salud mental de las niñas, niños y adolescentes y niñas, niños y adolescentes no acompañados refugiados y solicitantes de asilo ^13^ ^,^ ^14^. Si bien se han realizado algunos estudios con niñas, niños y adolescentes no acompañados migrantes mexicanos y centroamericanos en México y en EEUU, falta información respecto a las violencias experimentadas, principalmente en el origen, y los distintos problemas de salud mental que enfrentan ^15^ ^,^ ^16^ ^,^ ^17^.

Con relación a las estrategias de afrontamiento de las niñas, niños y adolescentes no acompañados ante las violencias, en Chile despliegan agencias limitadas, algo similar sucede con niñas, niños y adolescentes no acompañados centroamericanos en México. Algunas de las estrategias son: separación voluntaria de la familia, denuncia de los perpetradores, intercambio de productos por comida, cuidarse de los persecutores. La ausencia familiar es una fuente de resiliencia, que ocasiona independización forzada y la creación de redes de apoyo no familiares ^18^ ^,^ ^19^ ^,^ ^20^.

Este artículo busca responder a la pregunta: ¿Cómo se configura el *continuum* de violencia que enfrentan las niñas, niños y adolescentes no acompañados centroamericanos en sus comunidades de origen, durante el tránsito por México y en el destino en Estados Unidos, de qué manera estas experiencias afectan su salud mental y cuáles son las estrategias de afrontamiento que despliegan? Por ello, el objetivo es analizar las violencias experimentadas por niñas, niños y adolescentes no acompañados en las comunidades de origen en Centroamérica, durante el tránsito y el destino, explorando los problemas en salud mental presentes y las estrategias de afrontamiento implementadas.

## Material y métodos

Efectuamos un estudio cualitativo analítico con una muestra por conveniencia binacional. Entre junio del 2022 y junio del 2023 se hicieron entrevistas semiestructuradas en México y EEUU a niñas, niños y adolescentes no acompañados de origen centroamericano y a informantes clave que trabajaban con estas poblaciones. Con las niñas, niños y adolescentes no acompañados se exploraron los siguientes temas: contexto en el origen, viaje migratorio, necesidades de salud, estrategias de afrontamiento ante las adversidades y apoyo durante su tránsito y al llegar a México o EEUU. Con los informantes clave se abordaron los mismos temas, además de sus recomendaciones de políticas, estrategias para mejorar la salud y el bienestar de las niñas, niños y adolescentes no acompañados.

La investigación fue aprobada por la Comisión de Ética de la Universidad de California San Francisco (Proyecto CI: 21-35430) y del Comité de Ética en Investigación del Instituto Nacional de Salud Pública de México (Proyecto CI: 1785). Dado que el estudio involucró a niñas, niños y adolescentes no acompañados, se tomaron medidas éticas rigurosas para proteger su bienestar, privacidad y autonomía. Antes de cada entrevista, se explicó detalladamente en qué consistía el estudio, incluyendo sus objetivos, riesgos y beneficios, así como las medidas adoptadas para garantizar la confidencialidad y el uso seguro de la información. Se proporcionó un espacio para resolver dudas y se solicitó el asentimiento verbal informado de manera clara, adaptada al nivel de comprensión de las y los participantes. No se recopilaron datos personales identificables, y las transcripciones fueron anonimizadas eliminando nombres u otros elementos que pudieran permitir la identificación de las personas participantes. En el caso de las y los informantes clave, también se solicitó consentimiento verbal informado. En México, además se obtuvo el consentimiento de la dirección del albergue. Las niñas, niños y adolescentes no acompañados recibieron una tarjeta de regalo de USD 25.

En México, todas las entrevistas se realizaron en persona. En EEUU, con las niñas, niños y adolescentes no acompañados fueron en persona y con los informantes clave vía remota o en persona, según su disponibilidad. Estas duraron entre 30 y 90 minutos, se audiograbaron y transcribieron utilizando *Otter.ai* (para inglés) y *Amberscript* (para español). Se cotejaron todas las entrevistas y transcripciones para garantizar que estas fueran precisas.

Para el análisis temático ^21^ se desarrolló una lista inicial de códigos utilizando las principales preguntas de las guías de entrevista. Se crearon códigos adicionales basados en las transcripciones y las notas del trabajo de campo. Dos investigadores codificaron las dos primeras entrevistas para cada grupo (informantes clave y niñas, niños y adolescentes no acompañados), garantizando la coherencia entre codificadores, posteriormente se codificaron las restantes. Una prueba de confiabilidad entre codificadores obtuvo un valor kappa de Cohen promedio de 0,76. La codificación se realizó utilizando Dedoose (versión 9.0.54; https://www.dedoose.com). Se analizaron extractos codificados de todas las entrevistas utilizando matrices en Excel. Se compararon la respuesta entre participantes de diferentes grupos (tipo de informante, sexo de las niñas, niños y adolescentes no acompañados, y país de residencia). Los extractos se mantuvieron en el idioma original para la codificación y se tradujeron después del análisis. Un investigador bilingüe verificó todas las traducciones.

## Resultados

En total fueron entrevistadas 34 personas, 14 informantes clave (8 en México y 6 en EEUU) y 20 niñas, niños y adolescentes no acompañados (10 por país). De las niñas, niños y adolescentes no acompañados, la mayoría nació en Honduras (12), seguido por Guatemala (5), El Salvador (2) y Nicaragua (1). Seis migraron cuando tenían entre 8 y 12 años y catorce tenían entre 14 y 17 años. La edad promedio al momento de la migración entre las niñas, niños y adolescentes no acompañados entrevistados en México fue de 13,3 años, mientras que la de las niñas, niños y adolescentes no acompañados entrevistados en EEUU fue de 15 años, en ambos casos la edad promedio al momento de ser entrevistados fue de 16 años, habiendo pasado 3 años, en el caso de México, y 1 año en el caso de EEUU, entre el momento de la migración y el momento de la entrevista (Cuadro 1). 

**Cuadro 1:** Datos descriptivos de participantes.

LOCALIDAD Y ESTADO DE LA ENTREVISTA			TIPO DE ORGANIZACIÓN/INSTITUCIÓN		TIPO DE INFORMANTE CLAVE
MÉXICO					
Tapachula, Chiapas			OSC 1: ofrece asistencia legal y servicios sociales a niñas, niños y adolescentes no acompañados		Responsable área psicosocial
					Coordinadora de la sede de la organización
Tijuana, Baja California			Albergue de la Sociedad Civil 1 para niñas, niños y adolescentes no acompañados		Coordinadora de 3 albergues para niñas, niños y adolescentes no acompañados en frontera norte de México
Ciudad de México			Albergue de la Sociedad Civil 2 para mujeres y niñas, niños y adolescentes violentados		Directora
			Albergue de la Sociedad Civil 3 para población migrante en general		Enfermera
			Universidad privada		Investigadora y activista en el tema de niñas, niños y adolescente no acompañados
			Albergue de la Sociedad Civil 4 para niñas, niños y adolescentes no acompañados		Área de psicología
					Área de trabajo social
EEUU					
Fresno, California			OSC 1: ofrece servicios de abogacía a familias latinas		Directora de departamento
			Programa gubernamental de salud pública para adolescentes		Coordinadora en gestión de servicios y talleres
Stanford, California			Universidad/Departamento de medicina		Director de programas comunitarios sobre estrés y resiliencia en la infancia
Bahía de San Francisco, California			OSC 2: a favor de los derechos de los jóvenes excluidos de la educación, salud y bienestar social		Abogado
			Centro de salud de escuela primaria		Enfermera
			OSC 3: promotora para la inclusión de jóvenes vulnerables a través del deporte		Coordinador de programa
Total			12		14
NIÑAS, NIÑOS Y ADOLESCENTES NO ACOMPAÑADOS ENTREVISTADAS/OS EN MÉXICO					
PAÍS DE ORIGEN	SEXO	EDAD DE MIGRACIÓN Y EDAD EN LA ENTREVISTA		MOTIVO DE MIGRACIÓN	TIPO DE NO ACOMPAÑAMIENTO
Guatemala	Mujer	12	15	Violencia y rapto: migró contra su voluntad al ser raptada por un conocido de la familia	Migró al ser raptada por un hombre adulto quien abusó de ella sexualmente. Actualmente vive en un albergue para niñas, niños y adolescentes no acompañados en México
Honduras	Mujer	10	14	Pobreza: la mamá decidió migrar y ella migró con su mamá	Migró con su mamá, de quien se separó en México. Actualmente vive en un albergue para niñas, niños y adolescentes no acompañados en México
Honduras	Mujer	16	17 (A)	Búsqueda de oportunidades: acompañó a su prima mayor de edad a Guatemala y en el viaje decidieron cruzar a México, intentando llegar a EEUU	Migró con una prima a la que deportaron y actualmente vive en un albergue para niñas, niños y adolescentes no acompañados en México
Honduras	Mujer	16	17 (B)	Violencia: abuso sexual por un familiar en el país de origen	Migró sola. Actualmente vive en un albergue para niñas, niños y adolescentes no acompañados en México
Honduras	Mujer	16	17 (C)	Violencia y amenazas: integrantes de la delincuencia organizada la intentaron reclutar para delinquir	Migró sola. Actualmente vive en un albergue para niñas, niños y adolescentes no acompañados en México
El Salvador	Hombre	8	16	Violencia: integrantes de la delincuencia organizada lo intentan reclutar para delinquir	Migró con su mamá, papá y dos hermanos de quienes se separó. Actualmente vive en un albergue para niñas, niños y adolescentes no acompañados en México
El Salvador	Hombre	9	17	Violencia: su familia tenía problemas con integrantes de la delincuencia organizada y su abuelo fue asesinado por el Estado	Migró con un tío, una tía, primos y su hermana, de quienes se separó. Actualmente vive en un albergue para niñas, niños y adolescentes no acompañados en México
Honduras	Hombre	15	17 (A)	Violencia física y psicológica de familiares por su preferencia sexual homosexual	Migró con una prima a la que deportaron. Actualmente vive en un albergue para niñas, niños y adolescentes no acompañados en México
Honduras	Hombre	15	17 (B)	Abandono y negligencia familiar: madre ausente y negligencia por parte del papá	Migró solo. Actualmente vive en un albergue para niñas, niños y adolescentes no acompañados en México
Honduras	Hombre	16	17 (C)	Violencia y amenazas: el hermano de su novia estaba en la delincuencia organizada y lo amenazó para reclutarlo	Migró solo. Actualmente vive en un albergue para niñas, niños y adolescentes no acompañados en México
Total			10		
NIÑAS, NIÑOS Y ADOLESCENTES NO ACOMPAÑADOS ENTREVISTADAS/OS EN EEUU					
PAÍS DE ORIGEN	SEXO	EDAD DE MIGRACIÓN Y EDAD EN LA ENTREVISTA		MOTIVO DE MIGRACIÓN	TIPO DE NO ACOMPAÑAMIENTO
El Salvador	Mujer	16	18	Violencia: Acoso sexual y reunificación con su hermana	Migró sola. Actualmente vive con su papá y una hermana en EEUU
Guatemala	Mujer	17	18	Reunificación con hermanos y seguir estudiando	Migró sola. Actualmente vive con sus hermanos en EEUU
Honduras	Mujer	9	14	Pobreza y delincuencia. Temor de secuestro por bandas de delincuentes	Migró con su mamá, con la ayuda de un traficante. En el trayecto fue separada y reunificada con su mamá
Honduras	Mujer	12	16 (A)	Pobreza e inseguridad. Sin condiciones para acudir a la escuela	Migró con su mamá, con la ayuda de un traficante de personas
Honduras	Mujer	15	16 (B)	Pobreza y violencia intrafamiliar	Migró sola. Actualmente vive con su tía y prima en EEUU
Guatemala	Hombre	16	18 (A)	Pobreza y reunificación: ayudar económicamente a su familia y vivir con sus hermanos	Migró solo. Actualmente vive con sus dos hermanos en EEUU
Guatemala	Hombre	17	18 (B)	Pobreza y reunificación familiar	Migró solo. Actualmente vive con sus hermanos en EEUU
Guatemala	Hombre	18	18 (C)	Pobreza y reunificación familiar	Migró solo. Actualmente vive con familia extendida en EEUU
Honduras	Hombre	16	16	Pobreza y barreras para estudiar	Migró solo. Actualmente vive sin familiares en EEUU
Nicaragua	Hombre	14	14	Amenazas de muerte a su familia	Migró con su mamá y fueron separados en estación migratoria
Total			10		

OSC: Organizaciones de la Sociedade Civil.

Fuente: elaboración propia.

Nota: las letras entre parêntesis (A), (B), (C) son parte del identificador del informante, debido a que, hay dos informantes con el mismo sexo, la misma edad y la misma nacionalidad.

Se categorizó como niñas, niños y adolescentes no acompañados a quienes migraron teniendo menos de 18 años y que cumplieran alguna de las siguientes condiciones: (1) realizar el tránsito sin compañía de algún familiar/tutor; o (2) haberse separado de los familiares/tutores durante el trayecto o en el destino. Más de la mitad realizaron su viaje sin familiares/tutores (5 en México, 7 en EEUU). En EEUU, casi todos migraron con traficantes de personas y ninguno en México.

Al momento de la entrevista, en México la totalidad de las niñas, niños y adolescentes estaban no acompañados. Los tres que entraron al país con acompañantes (mamá, papá, hermanos, primos) se separaron debido a: (1) los familiares escaparon de las autoridades de migración o fueron deportados; o (2) sufrían abuso familiar, por lo que decidieron separarse de estos. Durante las entrevistas en EEUU, tres niñas, niños y adolescentes mencionaron que migraron con sus mamás, dos fueron separados de ellas en algún momento en los centros de detención. Se decidió dejar estos casos como parte del estudio.

La mayoría provenían de hogares monoparentales y experimentaron violencia y desintegración familiar, en Centroamérica generalmente vivían con la mamá y su pareja, con hermanos o medios hermanos. Al momento de las entrevistas, en México todos estaban en albergues, mientras que, en EEUU, con una excepción, habían logrado la reunificación familiar.

Se efectuaron 14 entrevistas con informantes clave (8 en México y 6 en EEUU), quienes pertenecían a Organizaciones de la Sociedad Civil (OSC) o albergues de la Sociedad Civil (7 en México y 3 en EEUU), instituciones académicas (3) y un agente de gobierno en EEUU (Cuadro 1).

### Violencia en el origen

De acuerdo a los informantes clave y las niñas, niños y adolescentes no acompañados, los motivos de la migración son variados, incluidos los económicos (falta de oportunidades y pobreza), la reunificación familiar y, principalmente, las múltiples violencias en el origen. Al acercase la adolescencia, a la violencia familiar se suma la violencia del crimen organizado, que busca el reclutamiento de niñas, niños y adolescentes para actividades delictivas. Si bien es algo más común en hombres, también sucede en mujeres. La violencia sexual por integrantes de la delincuencia y de la familia se mencionó adicionalmente en el caso de las mujeres. Así lo relata una psicóloga de una OSC quien mencionó que la gran mayoría de adolescentes ha vivido violencia familiar y violencia de las bandas criminales, y una joven hondureña, quien migró de 16 años después de ser amenazada al negarse a traficar drogas (Cuadro 2: Testimonio 1 y 2).

**Cuadro 2:** Testimonios de niñas, niños y adolescentes no acompañados centroamericanos e informantes clave sobre las violencias experimentadas en el origen, tránsito y destino, junio 2022- junio 2023.

1: Informantes clave, Psicóloga de OSC que ofrece asistencia legal y servicios sociales a niñas, niños y adolescentes no acompañados, Tapachula, México	“*Las niñas y mujeres han sido más violentadas en términos sexuales* (...) *la mayoría de la población* [niñas, niños y adolescente no acompañados] *con la que hemos contactado han vivido algún tipo de violencia física o doméstica. Casi que todos los adolescentes con los que hemos trabajado, vivieron ese tipo de situaciones en sus familias o de algún cuidador* (...) *la mayoría han vivido violencia secundaria de las pandillas, ¿no? han sido perseguidos, algunos de sus familiares han sido asesinados. Entonces, esta violencia emocional, psicológica, los niños particularmente también hablan mucho de la violencia física y las mujeres también, pero destacan más la violencia sexual…*”
2: Niñas, niños y adolescentes no acompañados, Hondureña en México, mujer, 16 (C) años	“*Yo me salí del colegio desde los 14 años, empecé a trabajar desde los 15* (...) *vendía mis hamburguesas* (...)*, me amenazaron, en broma me dijeron ‘mira te voy a dar una opción para que ganes más dinero, solo tienes que trabajar esto, solo tienes que meter esta bolsita* [con drogas]*, ahí y te damos el contacto a dónde vas a ir’* (...) *A los días, pasaron y ellos llegaban a mi casa y le decían a mi mamá,* (...) *venían armados y se ponían en una camioneta* (...)*. Tomaron una foto a mi hermanita, me dice, ‘mira a tu hermana, cómo entra tan feliz al colegio y tan alegre, es tan pequeña, ¿verdad?’* (...) *y le dije después, mira, si está bien, no te preocupes, yo lo acepto* (...)*. ‘¡No te vamos a estar esperando todo el tiempo!’, me dice, ‘así que, por favor, piénsalo rápido, sino yo voy a ser el que te va a apurar’, y levantó, de aquí, y me enseñó el arma.* (...) *Entonces, pasan los días y le comenté a mi mamá,* (...) *mire, me está pasando esto, me están amenazando y tengo mucho miedo, yo no quiero hacer eso,* (...) *se puso a llorar y se puso triste, me dijo que no aceptara, que no fuera a trabajar, entonces le dije a mi mamá ‘yo no sé qué hacer’. Tenemos dinero ahorrado, me dice, lo único que puedes hacer es que te puedes ir de aquí, de este país.* (...) *Mi mamá se mudó de la casa, el mismo día, salió en la madrugada ella también, vive en otro municipio, fue como me pude salir, porque si yo me regreso, a mí me matan,* (...) *Yo sentía mucho miedo, nunca llamé a la policía, iba a ser peor para mí, porque la policía se vende allá también, son delincuentes allá también…*”
3: Informantes clave, Coordinadora de albergues de la sociedad civil para niñas, niños y adolescentes no acompañados, Tijuana, México	“*Para nosotros el cambio más importante que hemos documentado es el incremento de perfiles que migran por motivos de violencia.* (...) *Tenemos muy presente el tema de personas víctimas por el narcotráfico, independientemente de donde vengan Nicaragua, Colombia* (...) *es el que más afecta a las personas y por lo cual incremento de chicas y chicos que corren riesgo de ser reclutados. Inclusive han estado adentro de alguno de estos grupos delictivos y están tratando de huir de ellos,* (...) *ha sido un nuevo descubrimiento para nosotros de que o te unes a la guerrilla o te unes al narco, y si no te unes, hay que aportar dinero para la manutención de estos movimientos. Si para nosotros también fue algo como muy reciente en este año, entonces ese ha sido el cambio más importante, registrar más personas que están huyendo por motivos de seguridad…*”
4: Niñas, niños y adolescentes no acompañados, Guatemalteca en México, mujer, 12 años	“*Era cuñado de mi prima. Y este tipo llegó a conocer a la familia* (...) *les dijo a mis papás que si lo podía ir a acompañar a la casa de mi prima, pero yo no quería ir* (...) *Entonces agarró otro camino y pasamos por montañas y pues como estaba chiquita* [12 años] (...) *Y ya no me pude regresar.* (...) *Cuando llegamos a la frontera de mi país con México, me dijo que veníamos para acá y a mí me daba miedo, pues no sabía qué hacer ¿no? Y, y las personas que me preguntaban que era de mí* (...)*. Y él así me tuvo, que era su hermana y abusó de mi tres veces...* *Como que rentaba* [en el Estado de México] *y ahí me encerró, me encerraba y se salía* (...)*. Y con las personas que le rentaron a él, pues se encariñaron mucho conmigo.* (...) *Y ya como les fui agarrando confianza. ¿Pues les dije no? Y ellos me escondieron en su casa y le dijeron que me había escapado* (...)*. Y me llevaron a la fiscalía y este, pues yo les conté todo, no era como un tipo secuestro para mí* (...) *y dijeron que me tenían que llevar al desarrollo Integral de la familia* [Sistema Nacional para el Desarrollo Integral de la Familia]... *Y me puede pasar lo peor, pero no lloro. Pero sí hay momentos que a veces exploto* (...) *me pueden ver feliz, le digo, pero no sabes lo que me ha de estar pasando.* (...) *Pero nunca les digo por qué estoy llorando.* (…). *Ya llega mi tutor en la mañana y a veces me dicen ‘¿Qué tienes?’ Y yo no tengo nada. Y ¿yo? Y de tanto que tu carita me dice todo, y yo ‘Ayyy’ y ya no aguanto y lloro, ¿no? y ya las abrazo y ya, ya me voy…*”
5: Niñas, niños y adolescentes no acompañados, Salvadoreño en México, hombre, 9 años	“*P. Dejaste de ver a tu mamá* *R. Sí y pues ya ahí empecé a vivir con mi tía,* [su mamá] *nos trataba a mí mal y a mi hermana, y pues mi tío empezó a violar a mi hermana* *P. No me digas, ¿Y tu papá sabía?* *-Sí* (...)*, mi hermana [había migrado a México] empezó a mandar cartas a las vecinas de atrás, entonces llegaron las del desarrollo integral de la familia* [Sistema Nacional para el Desarrollo Integral de la Familia] *y nos sacaron de ahí, como mi hermana era mayor, ella se podía hacer cargo de mí pues, y todavía tenemos contacto con mi papá, y luego nos hicieron contacto con mi mamá, pero no me interesaba hablar con ella ni con mi papá.* (...) *Y así pasaron como unos cuatro meses, llegó mi papá, dijo que nos iba a sacar* (...)*, y yo le dije que no, que no me iba a ir y se fue, pero vi que se iba con una camioneta de mi tío y pues qué bueno que no me fui* *P. ¿pero sí se llevó a tu hermana?* *R. Sí se la llevó, ella sí se quiso ir* *P. Pero entonces y, pero ¿sí están en EEUU?* *R. Sí, pero no con mi papá,* (...) *qué bueno que no me fui con él, porque mi hermana, la otra* [está en El Salvador]*, me contó de que mi otra hermana está en las calles de EEUU. Digamos que mi papá la dejó a su suerte y la dejó por las calles* (...)*, ella también tiene un problema.* *P. ¿Un problema como a nivel emocional, mental o algo...?* *R. Sí, mental, psiquiátrico, algo así...*”
6: Niñas, niños y adolescentes no acompañados, Hondureño en México, hombre, 15 (A) años	“*R. Estaba pensando, de si me venía para acá* (...) *lo pensaba porque mi mami me decía que no me quería* (...)*, me discriminaba por mi color de piel y me trataba de maricón. Entonces un día mi prima me dijo que se venía para México y si me quería venir con ella, yo le dije que sí y me vine para acá* (...)*. Hubo un tiempo que mi prima, no lo hacía por insultarme, le dijo a mi tío que yo era gay. Entonces mi tío me agarró del cuello y me dijo que me iba a matar, yo le tuve que decir que era mentira. Me soltó y me pegó y me dijo que si yo salía sí me iba a matar* *P. ¿Y eso cuántos años tenías?* *R. Tenía como diez...* *P. ¿Has tenido algún problema de salud?...* *R. Emocionalmente si me he sentido como que mal. Y también, lo que más me preocupa, es así como de que yo puedo estudiar, pero a mí no me se me quedan las cosas muy bien. Ok, Mhm, mhm. Y también por eso mi mamá me insultaba, me trataba de burro, de que no sabía nada* *P. ¿Y aquí has consultado a los psicólogos?* *R. Sí* (...)*, no me he sentido discriminado. Pero, a veces sí me siento como que mal porque no sé, por lo que me decía mi mami, me dan ganas de cortarme porque yo en un tiempo me cortaba. Por eso también me están dando medicamento para que no me pegue la ansiedad de cortarme...*”
7: Niñas, niños y adolescentes no acompañados, Nicaragüense en EEUU, hombre, 14 años	“*P. ¿Cuáles fueron las razones por las que dejaste Nicaragua?* *R. Es que mi mamá, a mí y a la familia pues en general nos amenazaron de muerte.* (...) *según dicen que fue que mi mamá que lo echó preso* [a la cárcel] *y nada que ver* (...)*. Y entonces pues nos vinimos por el miedo que nos mataran.* (...) *Y también por el gobierno que nos mandó amenazar. Sí, porque mi abuelo estuvo metido bastante en la política. Entonces por eso.* (...) *Mi abuelo ya es muerto, pues murió por una bomba, lastimosamente andaba pescando y le explotó una bomba. Saber de qué era...*”
8: Niñas, niños y adolescente no acompañados, Hondureña en México, mujer, 10 años	“*R. Nos quedamos parados porque estábamos esperando a los de atrás y vi como el hombre la violaba, y mi mamá, lo vimos, no sé quién no lo vio. Yo le dije a mi mamá lo está haciendo y mi mamá no podía* [hacer nada]*, porque también la llevaban a ella. O sea, nos tuvimos que quedar calladas.* (...) *Le cortaron los pedazos del cuerpo y lo colgaron, colgaron las manos y así cosas más feas. Si usted me pone una película de sangre, yo le voy a decir, ¡quíteme eso!, no me gusta la sangre porque me viene a la mente eso* *P. Tu mamá y tú… ¿cómo enfrentaron eso?* *R. Yo cuando quiero llorar, me echo a reír a carcajadas de la nada sin que me digan un chiste y yo quiero llorar* (...) *le decía, quiero llorar por lo de la muchacha, y decía ¡cállese! Yo sé que mi mamá actuaba muy fría, pero lo hacía para olvidar, para no volverlo a pensar.* (...) *Yo sé que es muy feo lo que usted vio, muy feo, porque yo también lo sentí, me dice mi mamá. Pero también tenemos que entender que así es la vida, cruel, me dice y que Dios tiene preparado algo para la persona que hizo eso. ¿Estás de acuerdo que nosotras no podíamos hacer nada? Sí mami, estoy de acuerdo* (...) *Te va a doler y vos vas a vivir con eso todo el tiempo en tu vida, es como una cicatriz que siempre la vas a llevar en tu piel. Lo que vos viste lo vas a llevar en tu mente, me decía...*”
9: Niñas, niños y adolescentes no acompañados, Guatemalteco en EEUU, hombre, 17 años	“*R. A mí lo que me dio miedo, cuando aparecieron los marinos atrás y nos apuntaron con el arma y al conductor también. Lo bueno que nos dejaron ir porque se fueron siguiendo al otro carro que iba delante de nosotros y creo que era un jefe del narco* (...) *y se escucharon un montón de balaceras y nos asustamos todos porque en el otro carro iban familiares de otros que iban en el carro* *P. ¿Sabes qué les pasó?* *- No, los dejaron ir porque pagaron. Como los soldados ahí en México son corruptos...*”
10: Informantes clave, Directora de albergue para mujeres y niñas, niños y adolescentes violentados, Ciudad de México	“*Ellas lo manifiestan,* (...) *dicen ‘no es que nos manosearon, nos metieron la mano, los de migración’, cuando van llegando al país y pues te van trasteando por todo el camino, te van tocando para ver que traes, ¿no? O sea, eso es parte, es una agresión sexual de la más agresiva...*”
11: Informantes clave, Enfermera de albergue para población migrante, Ciudad de México	“*Estaba acompañando el caso de un joven de 20 años que tenía 2 hermanos, conectados aquí [viviendo en varios albergues], luego el hermano de 20 años intentó suicidio y, de ahí se fueron,* (...) *pero como él estaba enfermo y era el tutor de los menores, no sabemos luego a donde se fueron, no tenemos noticias si ya están en EEUU o si los deportaron* (...) *La hermana, tuvo relaciones* [sexuales] *en la ruta migratoria para ganar dinero y esto impulsada por el propio hermano de 20 años, que tenía mucho celo de ella y para él era un, ella era un instrumento también,* (...) *incluso él se quedó 10 días hospitalizado aquí en el Fray Bernardino* (...) *el escapó, dejó a sus hermanos, incluso los medicamentos, entonces, sabíamos que él no tenía ninguna condición de cuidar de sus hermanos...*”
12: Informantes clave, Enfermera de albergue para población migrante, Ciudad de México	“*Este chico,* (...)*, él estaba solo, llegó menor, cumplió 18 años por aquí por México,* (...) *supimos que fue agresión dentro de la estación migratoria, lo golpearon fuertísimo y el quedó con secuelas* [físicas y psicológicas]*, para siempre* (...)*. Según, lo que tuvimos de información, fue de las autoridades. Fue a través de los propios migrantes que supieron, cuando la mamá puso en* [una red social de Internet] que, si sabían del chico, puso una foto, entonces, los propios migrantes, que estaban en EEUU, que estaban aquí por México, por [el sur del país] *mismo, es que ayudaron a identificar, dijeron ‘este chico está en la estación migratoria...’*”
13: Informantes clave, Investigadora y voluntaria en el tema de niñas, niños y adolescentes no acompañados, Ciudad de México	“*R. Entrar a EEUU como adolescente es mucho mejor que entrar de 18 años, ellos saben que quieren cruzar, la mayoría, ¿no? ¿cómo los van a dejar en el desarrollo integral de la familia* [Sistema Nacional para el Desarrollo Integral de la Familia] *11 meses?* (...) *Afectas todos sus derechos tanto en México como en EEUU, no son unos meses nada más, el tiempo es diferente para un adolescente* (...) *me decían, ‘perdí 11 meses en* (...) *Tapachula* (...) *me obligaron a vivir en el refugio porque era la única forma de transitar por México” y eso no es cierto porque existe una tarjeta de tránsito* (...) *por razones humanitarias de temas de violencia a adolescentes no acompañados, pero no se aplica porque el mismo Instituto Nacional de Migración la da por aquí y a unos kilómetros en otro Estado, le dice, no, esa no es válida y la rompen, entones no se aplica pero existe esa tarjeta,* (...) *lo que están haciendo el desarrollo integral de la familia* [Sistema Nacional para el Desarrollo Integral de la Familia] *y muchas OSC que le dice al adolescente que la única opción que tiene para transitar por México es pedir el refugio, ya cuando tienes tu tarjeta permanente ya puede moverte, aunque el aclara desde un principio que él no se quiere quedar en México* *P. Según nuestras entrevistas, al darle la calidad de refugiado ya no pueden acceder a ciertos documentos para entrar fácil a EEUU, ¿eso les impide o les obstaculiza?* *R. Aja, EEUU te pregunta si ya pediste refugio en otro país, entonces, la política de “tercer país seguro”, como ya estás seguro en otro país, entonces porqué vienes* (...) *yo creo que, con toda la migración, no solo con los adolescentes, México es lo que está haciendo* (...)*, hasta en Tijuana que ya es la frontera norte, todavía le preguntan a la gente ¿estás seguro que no quieres pedir el refugio en México? ACNUR,* [Alto Comisionado de las Naciones Unidas para los Refugiados] *es lo que paga, está pagando todo, para que la agencia de refugio, para que COMAR* [Comisión Mexicana de Ayuda a Refugiados]*, ¿no? todas las políticas, hay también recursos en medio y hay todo para detener a los migrantes aquí, como que están haciendo la frontera sur de EEUU, es Guatemala - México,* (...) *eso explica por qué hay tantos militares en la frontera sur...* *P. Hemos encontrado casos, nos han dicho, de menores que ya están en Monterrey y los mandan la Ciudad de México* *R. Es la deportación también, el derecho a la niñez dice que no puede ser deportado, que no puede ser devuelto a su país de origen, pero nadie, como no ponen el interés superior del niño, porque ellos toman la decisión por él, tampoco cumplen con la no deportación...*”
14: Niñas, niños y adolescentes no acompañados, Nicaragüense en EEUU, hombre, 14 años	“*R. Cuando entramos aquí a EEUU, nos separaron.* (...) *Nos contaron que había un policía racista pues que era un mexicano gordito y sí fue racista con nosotros* (...)*, nadie le dio orden de deportación, él solo fue, según dicen ellos, que el que pasó por la frontera a entrar de vuelta a México y los zumbó de vuelta a México y lo dejo ahí* *P. ¿Entonces pasaste la frontera hacia EEUU? Ahí estuvieron separados en un centro de detención de inmigrantes* *R. Sí. Demasiado helado (...) unos señores racistas. Nos tiraron la comida. Nos vivían diciendo cosas, pues, así como uno era recién llegado no sabía tanto inglés. Y hasta hace poco me di cuenta qué es lo que me decían, este, cosas así racistas* *P. ¿Y entonces estuviste separado de tu mamá tres días?* *R. Sí, y ella es una cárcel, es un lugar pues, de mujeres y yo en lugar de chavalos, pues de muchacho...*”
15: Informantes clave, Abogado, OSC sobre el derecho a la educación, salud y bienestar de los jóvenes, Oakland, EEUU	“*Hasta el primer momento en que un joven experimenta interacción con el gobierno, esencialmente lo ponen bajo custodia y lo envían a Aduanas y Protección Fronteriza, que es la cárcel. Y es un tipo de entorno que es completamente inapropiado para cualquier ser humano. Increíblemente inapropiado para los niños, especialmente para las niñas con alguna vulnerabilidad. Y según la ley, no deben estar allí más de 72 horas, eso ha sido violado cientos de miles de veces. Hemos documentado ampliamente las violaciones de eso. Tenemos declaraciones de niños que han estado en esas instalaciones durante días, semanas, meses.* (...) *Mientras estén bajo custodia, porque repito, eso es lo que más sé. Hay brechas absolutamente enormes. La salud mental al 1000% es simplemente abismal y, sinceramente, la salud física también. Tenemos queja tras queja de niños. Y a veces son cosas bastante mundanas, como que se fracturaron la muñeca y no fueron atendidos por un médico* [pronto] (...)*, pero al igual que las necesidades médicas básicas no se cubren con prontitud. Ya sabes, un niño fue transferido y sus gafas no fueron transferidas, y siguió preguntando y estuvo como tres meses sin gafas. Y él era muy, muy pobre, dije, literalmente puede ver que estás fingiendo que está recibiendo educación mientras está sentado en el aula y no puedes ver eso...*”
16: Informantes clave, Director de programas comunitarios universitarios en salud mental para niñas, niños y adolescentes, Stanford	“*Definitivamente, vimos, ya sabes, en relación con la política, vimos el aumento de la explosión de estas instalaciones de atención colectiva* [de niñas, niños y adolescente no acompañados]*. Entonces, miles de niños alojados en una gran instalación, que no fue diseñada para cuidar a los niños, ya sea un* [supermercado] *reconvertido o una ciudad de tiendas de campaña* (...) *Es simplemente imposible tener atención y protección individualizada para los niños, y reciben una evaluación rápida, pero de ninguna manera servicios integrales, especialmente cuando se trata de salud mental o tratamiento psicológico,* (...)*. También vi el tipo de prolongación del tiempo de detención* (...) *hasta varios meses, y algunos niños estaban bajo custodia y lo que se suponía que era una especie de custodia temporal, pero en ese tipo de limbo durante seis meses a un año o dos más, cierto, en algunos casos. Entonces, el doble golpe que supone estar en este tipo de centro de atención colectiva donde nadie puede recibir la atención individualizada que necesita por naturaleza.* (...) *cuanto más tiempo pasan en estas instalaciones, más se deterioran, ¿no? Su tipo de desesperanza y sentido de autonomía simplemente se erosiona.* (...) *Definitivamente escuchamos y vimos de primera mano el tipo de daño psicológico de este tipo de, supongo, los impactos de estas políticas...*”
17: Informantes clave, Enfermera de centro de salud de escuela primaria, Oakland	“*Yo diría que de los seis jóvenes que he entrevistado, y había algunas mujeres, aproximadamente la mitad con edades comprendidas entre los 14 y los 22 años, eran suicidas, o habían sido agudamente suicidas o habían pensado en el suicidio, o habían intentado hacerse daño a sí mismos. Y eso es, ya sabes, eso es un número muy alto...*”
18: Informantes clave, Investigadora y voluntaria en el tema de niñas, niños y adolescentes no acompañados, Ciudad de México	“*Son jóvenes, ganadores de vida, muy listos y listas porque ellos saben por dónde, muy hábil, saben cómo se mueven, se comunican, tienen red de apoyo entre varios, como lo que cruzaron, como que avisan dónde están,* (...) *hacen grupos de amigos de la misma edad porque tienen la misma historia,* (...) *ellos saben que al ser menor no acompañado o adolescente no acompañado, tienen más derechos, cuando les conviene grita ‘yo soy adolescente, no acompañado’, ‘todavía no cumplo los 18’ lo usan como estrategia, ¿no? Y también me ha tocado una joven de 15 años que es mamá de una niña de meses, también en Tijuana y cada vez que se acercaba migración ella decía ‘madre soltera y menor de edad’* (...) *tienen estrategias para sobrevivir...*”
19: Niñas, niños y adolescente no acompañados, Guatemalteco en EEUU, hombre, 15 años	“*P. Llegando acá* [EEUU]*, cuando te reuniste con tu prima, ¿cómo fue?* *R. Pues feliz,* (...) *Tenía rato de no hablar con ella. O sea, cuando yo me vine, yo no le llamé que venía, solo me vine y pues sí, se sintió bien* (...)*. Mi prima me dijo, mira, aquí está esto, aquí está lo otro. Si necesitas algo, tienes problemas, pues habla. Nosotros estamos aquí. De hecho, he tenido algunos problemas, no graves, entonces, pues sí, me ha ayudado y ha estado al pendiente de mí...* *P. Cómo te sientes como comparando cómo te sentías esos primeros días a cómo te sientes ahora, ¿qué dirías?* *R. Mucho mejor* (...)*, por ejemplo, llego a la casa y pues mi prima está ahí, todos. Entonces, cenamos juntos pues, cosa que yo no tenía en Guatemala, cenar con alguien o comer con alguien a la par. Y cada, cada vez que nos sentamos pues hay algo que platicar. Yo le cuento algo nuevo, ella cuenta algo nuevo. Pues, hay comunicación. Entonces no es tanto el aburrimiento en la casa* *P. Ya, hay como, se siente ese ambiente familiar* *-Ajá, o si alguien tiene un problema o hay problemas en la casa, pues se platica y todo eso...*”

OSC: Organizaciones de la Sociedad Civil.

Fuente: elaboración propia.

Nota: identificadores: informante clave, niño, niña o adolescente, nacionalidad o institución en la que trabaja, edad al momento de la migración. Nota: las letras entre parêntesis (A), (B), (C) son parte del identificador del informante, debido a que, hay dos informantes con el mismo sexo, la misma edad y la misma nacionalidad.

Al enterarse de la situación, principalmente las madres, tomaron la decisión de que las niñas, niños y adolescentes migraran rumbo a EEUU, ellas o algún otro familiar facilitaron el dinero para el tránsito migratorio. Esta decisión es inmediata, buscando que no se reclute y violente a las niñas, niños y adolescentes o a sus familiares, quienes suelen cambiarse de lugar de residencia en el origen. El testimonio de la joven hondureña mencionada anteriormente retrata estas circunstancias, su madre le indicó que la única opción que le quedaba era migrar con ayuda de unos ahorros que ella tenía y ambas migraron el mismo día, la joven rumbo a EEUU y la mamá rumbo a otro municipio (Cuadro 2; Testimonio 2). No todos las niñas, niños y adolescentes no acompañados evitaron reclutamiento, algunos trabajaron para ellos hasta que pudieron escapar y migrar, como afirma la coordinadora de un albergue en México (Cuadro 2; Testimonio 3). Una niña, niño y adolescente no acompañados guatemalteca fue secuestrada cuando tenía 12 años con engaños, por un conocido de su familia que pertenecía a una red de trata de personas, y logró escapar en la Ciudad de México con ayuda de unos vecinos (Cuadro 2; Testimonio 4).

También fue un motivo de migración de las niñas, niños y adolescentes la violencia de las madres, sus parejas, los tíos o amistades de la familia. Además del abandono, los padres ejercieron violencia verbal y el encubrimiento de los agresores de sus hijas e hijos, tal como lo narra un joven salvadoreño que migró a los 9 años junto con su hermana, bajo el cuidado de su tía y tío, quienes los trataban mal, abusando sexualmente el tío de su hermana, y un joven hondureño que a los 15 años fue amenazado de muerte por su tío al sospechar su orientación sexual (Cuadro 2; Testimonios 5 y 6).

Los funcionarios públicos también ejercieron violencia. Se mencionó como motivo de la migración en Nicaragua y Honduras la persecución política de algún familiar, según lo describe un joven de 14 años proveniente de Nicaragua, en donde el gobierno amenazó a su familia por motivos políticos (Cuadro 2; Testimonio 7). Además, las fuerzas armadas no contienen la violencia del crimen organizado e incluso se llegan a coludir con ellos, por lo que denunciar puede ser peligroso (Cuadro 2; Testimonio 2).

### Violencia en el tránsito

Esta se ejerce principalmente por el crimen organizado. Las niñas, niños y adolescentes pueden experimentar violencia directa o ser testigos de cómo se ejerce hacia otras personas migrantes a través de asaltos, secuestros, violaciones, asesinatos y enfrentamientos armados. Cuenta de esto da el testimonio de una menor hondureña que, a los 10 años, junto con su mamá, presenció la violación, el asesinato y la mutilación de una mujer. Mientras que un joven de Guatemala de 17 años y el grupo de migrantes con el que venía, fueron detenidos por los marinos durante su tránsito por México, quienes les apuntaron con sus armas, para posteriormente perseguir a unos delincuentes, desatándose una balacera (Cuadro 2; Testimonio 8 y 9).

En Centroamérica y México las autoridades también ejercen violencia contra las poblaciones migrantes, extorsionándolas, al pedir dinero para permitirles continuar su viaje. Se identificó violencia sexual hacia las mujeres por parte de hombres del crimen organizado (violación y asesinato), familiares (trata de personas o encubrimiento de perpetradores) y autoridades (tocamientos indebidos), tal como lo afirman la directora de un albergue en la Ciudad de México, respecto al “manoseo” que ejercieron los agentes de migración, y la enfermera de otro albergue en la misma ciudad, quien narra cómo un hermano presionó a su hermana menor para que tuviera relaciones sexuales a cambio de dinero (Cuadro 2; Testimonios 10 y 11).

### Violencia en el destino

En México, en ocasiones, las autoridades pusieron a las niñas, niños y adolescentes no acompañados en centros de detención para población adulta. La enfermera del albergue en la Ciudad de México mencionó que, en una estación migratoria del sur del país, un personal del lugar golpeó muy fuerte a un niña, niño y adolescente no acompañado, dejándole secuelas físicas y mentales a largo plazo (Cuadro 2; Testimonio 12). A pesar de ser niñas, niños y adolescentes no acompañados, también se mencionaron deportaciones contra su voluntad.

En la gran mayoría de los casos, las niñas, niños y adolescentes no acompañados buscaban llegar a EEUU, a los que fueron detenidos en México se les truncó su proyecto migratorio. En EEUU se otorgaba con mayor facilidad refugio a niñas, niños y adolescentes no acompañados que no lo habían solicitado en otro país, pero distintas instancias en México les hacían creer que les convenía que les otorgaran el refugio en territorio mexicano, lo que obstaculiza migrar a EEUU. Respecto a los trámites para su protección, los agentes del Instituto Nacional de Migración (INM) llegaban a no reconocer la tarjeta de tránsito por el país, tal como lo afirma una investigadora del tema entrevistada, al señalar que en el sur del país el INM otorga esta tarjeta y más adelante dicen que no es válida y ellos mismos la rompen (Cuadro 2; Testimonio 13).

Respecto a la violencia de las autoridades en EEUU, en los centros de detención se dio maltrato verbal y racismo de los guardias; la reclusión a migrantes se da por edades y sexo, lo que ocasionó la separación temporal de las niñas, niños y adolescentes que iban con sus madres. Las instalaciones inadecuadas ocasionan hacinamiento, frío, falta de ventilación e iluminación. Se mencionó que las estancias en los centros pueden ser prolongadas, contraviniendo la ley; además, es inadecuada la atención médica y de su salud mental. Por último, las niñas, niños y adolescentes no acompañados no tienen la suficiente información sobre los procedimientos para obtener el refugio. Así lo ejemplifica el relato de un joven originario de Nicaragua de 14 años, separado de su madre en el centro de detención, y las experiencias de dos expertos en el tema trabajando en OSC en los EEUU, uno de los cuales afirma que miles de niñas, niños y adolescentes no acompañados han sido alojados en instalaciones inadecuadas, donde no se puede dar la atención y protección necesarias y en las que llegan a permanecer hasta por meses o años (Cuadro 2; Testimonios 14, 15 y 16).

**Figura 1:**
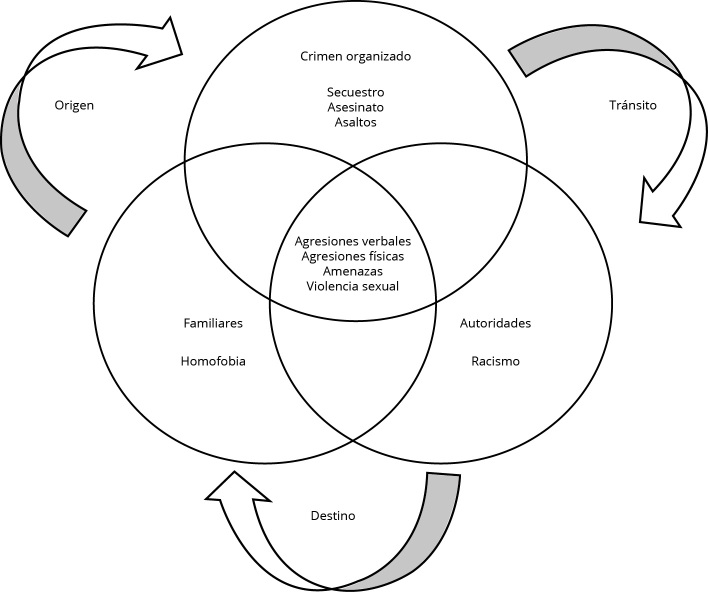
*Continuum* violencia experimentada por menores niñas, niños y adolescentes no acompañados migrantes.

El siguiente diagrama busca sintetizar la información más relevante con respecto al *continuum* de violencia de las niñas, niños y adolescentes no acompañados entrevistados en el estudio (Figura 1). Cada uno de los tres círculos (conjuntos), representa a los distintos actores sociales que ejercen la violencia (familiares, crimen organizado y autoridades), y en las intersecciones de los conjuntos aparecen las violencias que han experimentado las niñas, niños y adolescentes no acompañados en relación a los actores.

Fuente: elaboración propia.

### La salud mental de las niñas, niños y adolescentes no acompañados

Los distintos padecimientos en salud mental que mencionaron las niñas, niños y adolescente no acompañados, pudieran estar relacionados a las violencias experimentadas en el origen (secuestro, abuso sexual, homofobia, violencia verbal) y durante el trayecto migratorio, tal como se puede leer en las experiencias traumáticas experimentadas por las niñas, niños y adolescentes no acompañados al presenciar asesinatos o estar en medio de balaceras (Cuadro 2; Testimonios 4, 5 y 6). Adicionalmente, la respuesta de las autoridades en forma de centros de detención puede contribuir a estos padecimientos. Los informantes clave señalan el tratamiento inadecuado y la pobre atención que hoy día las niñas, niños y adolescentes no acompañados reciben, describiendo trato violento hacia las niñas, niños y adolescentes no acompañados ejercido por las propias autoridades migratorias (Cuadro 2; Testimonio 12).

Tanto las niñas, niños y adolescentes no acompañados como los informantes clave describen que en los centros de detención también se ejerce violencia, lo que, lejos de solucionar, contribuye a las consecuencias en salud mental de los jóvenes. Así lo explica un experto en salud mental en EEUU al mencionar como aumenta el daño psicológico de las niñas, niños y adolescentes no acompañados a medida que pasan más tiempo en estos lugares (Cuadro 2; Testimonio 16). En estos centros de detención, las niñas, niños y adolescentes no acompañados no tienen libertad y están en la incertidumbre por la falta de claridad sobre el proceso, a la vez que las detenciones en México, que les impiden llegar a EEUU, les generan emociones conflictivas.

Los informantes clave mencionan los retos que las autoridades tienen para atender su salud mental, como son las barreras para que las niñas, niños y adolescentes no acompañados hablen de sus experiencias, sea por miedo a la estigmatización, por no querer hablar de sus emociones con otros o como una estrategia de olvido, además de que se podría dar una revictimización, y afirman que muchos tienen ideas suicidas, en consonancia con el relato de una enfermera en EEUU, la cual mencionó que muchas niñas, niños y adolescentes no acompañados habían pensado o intentado suicidarse (Cuadro 2; Testimonio 17). Mencionan la urgencia de abordar de forma integral su salud mental, para no perpetuar el círculo de la violencia, donde pueden normalizarla o ejercerla contra otras personas.

### Estrategias de afrontamiento de las niñas, niños y adolescentes no acompañados 

A pesar del *continuum* de violencia que experimentan las niñas, niños y adolescentes no acompañados en los contextos de origen, tránsito y destino, estos adolescentes desarrollan diversas estrategias de afrontamiento para protegerse o superar las consecuencias de dichas violencias. Estas estrategias, lejos de ser pasivas, reflejan agencia, resiliencia y capacidad adaptativa frente a contextos altamente adversos.

En el país de origen, algunos adolescentes iniciaron la migración como una forma de escapar de situaciones de violencia directa, como amenazas, abusos familiares, violencia de género o violencia comunitaria, como se observa en los testimonios de la mujer que migró para no ser reclutada por las bandas criminales o el hombre que migra por miedo a ser asesinado (Cuadro 2; Testimonios 2 y 7). Durante el tránsito, implementaron estrategias como denunciar perpetradores o alejarse de ellos, crear redes de apoyo con pares u otros migrantes, o con personal de OSC, mantener contacto con familiares a través de redes sociales como WhatsApp y Facebook y replantear su proyecto migratorio al decidir quedarse en México y solicitar asilo en lugar de continuar su viaje hacia EEUU. Una investigadora en el tema menciona el conocimiento que tienen los propios jóvenes de sus derechos, recordando el caso de una adolescente con una hija de meses, quien le informaba a las autoridades de migración que era menor de edad y mamá, sabiendo que un niña, niño y adolescente no acompañado tiene más derechos de protección (Cuadro 2; Testimonios 4, 5 y 18). En el destino, otras estrategias incluyen continuar con sus estudios y reintegrarse con familiares con los que mantenían una buena relación, como lo describe un joven guatemalteco que logró reunificarse con su familia en EEUU (Cuadro 2; Testimonio 19).

Estas acciones demuestran que las niñas, niños y adolescentes no acompañados no pueden ser concebidos únicamente como víctimas pasivas. A pesar de las múltiples formas de violencia que enfrentan, conservan recursos y motivaciones personales, sociales y simbólicas que les permiten resistirse, reconstruirse y proyectar un futuro distinto, lejos de las violencias.

## Discusión

A lo largo del documento se aporta nueva y variada evidencia sobre la existencia de un *continuum* de violencia sistemática contra poblaciones migrantes centroamericanas menores de edad. Tanto en este estudio como en la investigación de Ruíz & Varela ^11^, las violencias experimentadas por niñas, niños y adolescentes no acompañados inician en los países de origen, y persisten en los países de tránsito y destino, bajo un esquema patriarcal, en donde su edad y género incrementan las vulnerabilidades en las dinámicas desiguales con personas adultas (familiares, del crimen organizado y de las autoridades) ^10^. En el presente documento, la mayoría de las violencias fueron experimentadas indistintamente por niñas, niños y adolescentes no acompañados, hombres y mujeres, pero la violencia sexual sucedió únicamente en el caso de las mujeres. En varias ocasiones estas violencias tuvieron afectaciones a su salud mental, incluso psiquiátricas, las cuales podrían relacionarse con ideaciones e intentos suicidas. Esta exposición a múltiples formas de violencia podría incrementar los riesgos de violencias posteriores, con impactos psicobiológicos menos reversibles y mayores niveles de ansiedad y otras sintomatologías traumáticas ^18^.

Aunque las niñas, niños y adolescentes no acompañados mencionaron como motivos de la migración la precariedad económica en el origen y la búsqueda de la reunificación familiar en EEUU, la violencia fue el motivo más mencionado, pudiendo catalogarse varios de estos casos como migración forzada, lo que también se sustenta por la condición de refugiados que les otorgaron las autoridades de EEUU y México.

A pesar de que en los países de origen y en México existe un reconocimiento jurídico del interés superior de la niñez, los resultados constatan no solo una insuficiente e inadecuada protección gubernamental hacia las diversas violencias ejercidas por familiares y el crimen organizado, sino que también coloca a las autoridades encargadas de salvaguardar a las niñas, niños y adolescentes no acompañados como una fuente de violencia, tanto en Centroamérica como en México y EEUU.

Si bien las niñas, niños y adolescentes no acompañados experimentaron estas diversas adversidades, es incorrecto percibirlos como víctimas pasivas, ya que desarrollaron estrategias para afrontar el *continuum* de violencia, como migrar, alejarse de la familia, denunciar al perpetrador y conformar nuevas redes sociales, resultados que coinciden con los encontrados por Zamora ^19^ y Corona-Maioli et al. ^20^. El reconocimiento de la agencia en estas poblaciones es trascendental y un cambio de paradigma, al reconocerlos como actores sociales y no como meros objetos de protección o cuidado ^18^.

A pesar de que el objetivo fue analizar las violencias experimentadas por niñas, niños y adolescentes no acompañados en el origen, tránsito y destino, en futuras investigaciones al respecto, sería importante incorporar la perspectiva de la interseccionalidad, ampliamente utilizada en los últimos años en estudios de género. En el caso de mujeres migrantes venezolanas en Brasil, se ha documentado la intersección entre la violencia de género y la xenofobia. Si bien se han realizado pocos estudios con niñas, niños y adolescentes, en alguno de ellos se ha evidenciado la interacción del género, la raza, las desigualdades sociales, el estigma y los prejuicios a las enfermedades mentales ^22^ ^,^ ^23^. Aunque no se exploró la interseccionalidad en las entrevistas, pareciera que en las niñas, niños y adolescentes no acompañados migrantes interactúan sus condiciones de no acompañamiento, el ser menores de edad, la irregularidad migratoria, el género, entre otras características, para explicar el contexto de vulnerabilidad donde se da el *continuum* de violencias experimentadas.

## Conclusiones

La presente investigación evidencia la compleja realidad que enfrentan las niñas, niños y adolescentes no acompañados centroamericanos en su proceso migratorio hacia México y EEUU, resaltando el *continuum* de violencias a las que están expuestos en sus países de origen, durante el tránsito y en los lugares de destino. La violencia estructural, familiar e institucional se manifiesta de diversas maneras, afectando no solo su integridad física, sino también su salud mental y bienestar emocional.

Los hallazgos subrayan que, a pesar del reconocimiento jurídico del interés superior de la niñez en los países de origen y en México, las medidas de protección gubernamentales resultan insuficientes o incluso generadoras de nuevas formas de violencia contra esta población vulnerable. La detención de las niñas, niños y adolescentes no acompañados en centros inadecuados, la separación familiar forzada y la falta de información sobre sus derechos y procesos de refugio contribuyen a su revictimización y afectan su estabilidad emocional.

Sin embargo, este estudio también destaca la capacidad de resiliencia y agencia de las niñas, niños y adolescentes no acompañados, quienes implementan estrategias activas de afrontamiento tales como la migración para escapar de las violencias, la denuncia de agresores, la creación de nuevas redes de apoyo y la continuidad educativa. Estas estrategias no deben ser invisibilizadas, sino reconocidas y potenciadas desde el diseño de políticas públicas. En este sentido, se hace necesario transitar hacia un enfoque más proactivo, integral y centrado en la escucha de las niñas, niños y adolescentes no acompañados, que promueva estrategias como la creación de espacios seguros de escucha activa donde se validen sus experiencias sin prejuicios, el fortalecimiento de la atención psicosocial y en salud mental, con equipos capacitados en trauma, migración y niñez, la capacitación del personal de diversas instituciones de gobierno, con enfoque en derechos humanos e infancias, la participación activa de las niñas, niños y adolescentes no acompañados en la construcción de soluciones, reconociéndolos como sujetos de derechos y la coordinación efectiva para garantizar rutas de protección y atención sostenibles y respetuosas de los derechos de las infancias migrantes. Un abordaje integral y coordinado entre los países involucrados es crucial para garantizar su derecho a una vida segura y digna.

## Data Availability

Los datos de la investigación están disponibles previa solicitud al autor correspondiente.
